# Induction of obesity impairs reverse cholesterol transport in *ob/ob* mice

**DOI:** 10.1371/journal.pone.0202102

**Published:** 2018-09-14

**Authors:** MyNgan Duong, Kiyoko Uno, Victoria Nankivell, Christina Bursill, Stephen J. Nicholls

**Affiliations:** 1 Heart Health, South Australian Health and Medical Research Institute, Adelaide, South Australia, Australia; 2 Department of Cell Biology and Cardiovascular Medicine, Cleveland Clinic Foundation, Cleveland, Ohio, United States of America; University of Milano, ITALY

## Abstract

**Objectives:**

Obesity is an independent risk factor for cardiovascular disease. Reverse cholesterol transport (RCT) is an important cardioprotective mechanism. This study aimed to investigate RCT changes in a murine model of obesity.

**Methods:**

*Ob/ob* and control mice were injected with [^3^H]-cholesterol-labelled macrophages and cholesterol accumulation quantified after 48 h. *Ex vivo*, cholesterol efflux and uptake were determined in hepatic and adipose tissues.

**Results:**

*Ob/ob* mice had more labelled cholesterol in their plasma (86%, p<0.001), suggesting impaired RCT. SR-BI-mediated cholesterol efflux was elevated from *ob/ob* mice (serum, 33%; apoB-depleted plasma, 14%, p<0.01) and HDL-c were also higher (60%, p<0.01). *Ex vivo* it was found that cholesterol uptake was significantly lower into the livers and adipose tissue of *ob/ob* mice, compared to non-obese wildtype controls. Furthermore, *ex vivo* cholesterol efflux was reduced in *ob/ob* liver and adipose tissue towards apoA-I and HDL. Consistent with this, protein levels of SR-BI and ABCG1 were significantly lower in *ob/ob* hepatic and adipose tissue than in wildtype mice. Finally, labelled cholesterol concentrations were lower in *ob/ob* bile (67%, p<0.01) and faeces (76%, p<0.0001).

**Conclusion:**

Obesity causes impairment in RCT due to reduced plasma cholesterol uptake and efflux by hepatocytes and adipocytes. A reduction in the capacity for plasma cholesterol clearance may partly account for increased CVD risk with obesity.

## Introduction

Obesity is a no longer a condition restricted to Western populations but is now recognised as a world-wide pandemic. Obesity is a risk factor for numerous chronic diseases including cardiovascular disease, diabetes and cancer [1–3].

Adipose tissue is an endocrine organ as it produces cytokines, known as adipocytokines, which have autocrine as well as paracrine affects and contribute to the body’s inflammatory status [4]. In obesity, adipocytes become dysfunctional, likely due to excessive lipid accumulation and results in excess free fatty acid (FFA) and pro-inflammatory cytokine production. These adipocytokines have downstream effects such as polarisation of resident macrophages to an inflammatory phenotype, changing adipocyte function and recruitment of more macrophages to adipose tissue [5]. This results in a cascade of pro-inflammatory cytokine production/secretion and if left unchecked may initiate and/or contribute to atherogenesis [6].

Epidemiological and pre-clinical studies show that high density lipoproteins (HDL) are cardio-protective. A consistent inverse relationship exists between plasma HDL cholesterol (HDL-c) and cardiovascular risk. Its anti-atherosclerosis properties include a role in cellular cholesterol removal and hepatic clearance via reverse cholesterol transport (RCT), anti-inflammatory and anti-oxidant properties as well as vasodilatory and anti-coagulant effects. Despite this, large clinical trials using HDL raising therapies are yet show consistent benefit [7,8]. This has prompted the notion that HDL may become dysfunction under certain metabolic milieu [6]. For example, serum from juvenile rheumatoid arthritis patients has lower cholesterol efflux potential [9] and HDL from diabetic patients and patients with renal disease have decreased RCT capacity [10,11]. This suggests that HDL function may also be negatively impacted upon by obesity, an inflammatory and metabolic disorder.

The RCT pathway involves the efflux of cholesterol from cells to HDL, including from macrophages within atherosclerotic plaques, and then the transport of HDL to the liver for processing before removal of the cholesterol in the bile and faeces. Cholesterol is essential to cell survival but excess cellular cholesterol is detrimental to cell functionality and integrity [11]. Therefore, cellular cholesterol efflux and RCT are critical for correct cellular function, particularly plaque macrophages as lipid accumulation leads to foam cell formation and fatty streak development. Changes in cholesterol efflux and RCT in the context of obesity are, however, yet to be investigated.

The aim of this study was to investigate the effect of obesity on RCT. Using the *ob/ob* mouse, in which a mutation in the leptin gene results in excessive appetite and obesity from 6 weeks of age, it was found that obesity had a significant negative impact on the movement of cholesterol from the circulation to the faeces. There was an impairment in the transport of cholesterol both in and out of hepatic and adipose tissues. This resulted in the retention of cholesterol in the plasma, providing an explanation, at least in part, for the increased risk of CVD in patients with obesity.

## Methods

*Ob/ob* and wildtype littermate control mice (n = male 14 x 8 week old mice; Jackson lab, Bar Harbor, ME, USA) were fed a standard chow diet. All studies were conducted in accordance with the Guide for the Care and Use of Laboratory Animals (National Research Council, 2011) and the Cleveland Clinic IACUC. This study was approved by the Cleveland Clinic Foundation IACUC. Mice were anesthetized with isoflurane as required and were humanely killed with an overdose cocktail of ketamine/xylazine (300mg/kg and 30mg/kg respectively).

### Quantifying percent body fat

The quantification of percent body fat in mice (n = 3 for each group) was determined by measuring total body water using heavy water (D_2_O) with the assumption that adipose does not take up D_2_O. Pre-dose plasma samples were taken prior to I.P. administration of the D_2_O (0.15 g/kg body weight) and then post-dose 3 h later. Mice abstained from food and water consumption during this period. Total body water was then calculated from the dilution of the D_2_O in the blood using isotope ratio mass spectrometry. Adipose mass was determined as the difference between total body mass and total body water [12,13].

### *In vivo* reverse cholesterol transport

This procedure is based on the method detailed by Zhang *et*. *al*. (2003) [14]. Briefly, macrophages (J774 cells) were tracer-labelled using [^3^H]-cholesterol (2.5 μCi/ml media; Perkin-Elmer, Waltham, MA, USA) in 1% FBS RPMI media with penicillin/streptomycin (P/S) for 24 h before being washed with serum-free media. The cells were then incubated overnight in 0.2% BSA RPMI with P/S. The cells were concentrated, after being washed twice with serum-free media, into a volume of 0.5 ml per mice and injected (approximately 10x10^6^ cells/ml; 10x10^6^ cpm/ml) into the animals intraperitoneally. Plasma was collected at 0, 6, 24 and 48 h after the injection and quantitated for [^3^H]-cholesterol using liquid scintillation counting. At 48 h, the animals are humanely killed and bile, liver, visceral fat and faeces were collected and [^3^H]-cholesterol was quantitated in these tissues.

### [^3^H]-cholesterol extraction from tissue

Tissues including liver and adipose were minced and lipids were extracted using the Bligh-Dyer method in a known mass of tissue [14]. Faeces (about 100 g) were also homogenised and lipids extracted in chloroform: methanol mixture (2:1). The solvent layer was dried down and reconstituted in toluene and [^3^H]-cholesterol then quantified using liquid scintillation counting. The amount of labelled cholesterol was adjusted to individual total plasma volume, normalised to tissue weight for liver and adipose tissue and total bile volume before calculation as a percentage of injected dose.

### Cholesterol efflux assay

Cellular cholesterol efflux assays were performed as reported elsewhere [15]. Briefly, cells were plated and trace labelled with [^3^H]-cholesterol (1.2 μCi/ml) in 1% FBS media for 24 h. Cells were equilibrated in 0.2% BSA media overnight before serum (2% v/v) and apolipoprotein B (apoB)-depleted serum (1.8% v/v) were incubated for 4 h. The media were quantitated for the amount of [^3^H]-cholesterol that was effluxed from the cells by liquid scintillation counting. This was expressed as percent efflux by dividing by total label uptake in the cells over a period of 4 h. J774 cells [16] were used to look at ABCA1-mediated cholesterol efflux, while Fu5AH [17] and BHK cells [18] were used to address SR-BI- and ABCG1-medaited efflux respectively. BHK was stably transfected with human ABCG1 cDNA under the control of mifepristone (10 nM) using the GeneSwitch system [18].

### *Ex vivo* liver and adipose cholesterol efflux assay

The *ex vivo* cholesterol assay was based on that published by Chung *et*. *al*.[19]. Briefly, fresh adipose or liver tissue (0.1–0.2 g) was finely chopped up (~2 mm pieces) and incubated for 24 h in serum-free DMEM F1/2 Ham. The tissue pieces were then divided up into roughly equal amounts in 12-well plates (n = 4 *ob/ob* and 8 control for liver tissue or 12 control mice due to discrepancy in liver and adipose mass respectively). The tissue culture was then washed carefully before being labelled with [^3^H]-cholesterol (3.0 μCi/ml) for 24 h. Prior to the efflux period of 4 h, the culture was equilibrated for 1.0 h before HDL (50 μg/ml), apoA-I (10 μg/ml) or media alone were added as cholesterol acceptors. The media was then collected and cell debris removed before the amount of radioactivity determined by liquid scintillation counting. The amount of radioactivity in the cells were also quantified after lipid extraction with isopropanol. The percent efflux was calculated as the amount of radioactivity in the media, as a percent of the total amount of radioactivity in the cell + media.

### Cholesterol uptake assay

The hepatic and adipose cholesterol liver uptake was determined in the same manner as the *ex vivo* cholesterol efflux assay up until the equilibration period. Instead, the cultures were washed and lipids extracted with isopropanol over 24 h and then the radioactivity quantified with liquid scintillation counting. The protein concentration was determined, for each tissue culture in each well, by homogenising the tissue in RIPA buffer to form a lysate and then protein was measured by using the BCA assay. The percentage of cholesterol uptake was determined as the percent of disintegrations per minute (dpm) in the culture relative to the total amount of dpm in the original labelling media and normalised to the protein concentration determined in the respective wells.

### Bile compositional analysis

Bile recovered from RCT was used to determine the compositional endpoint of the [3H]-cholesterol, loaded in the injected macrophages, using a previously published method [20] to separate neutral sterols from bile acids (BA). Briefly, bile was mixed with 0.4 ml of methanol, before the addition of 2.0 ml of petroleum ether (Sigma, St. Louis, MO, USA). This mixture was agitated for 4 min and then centrifuged for 20 min at 2000 rpm at room temperature. Both fractions were collected (ether fraction contains the neutral sterols while the hydrophilic fraction contains the BA), dried down and resuspended for scintillation counting. The percentage of composition was determined as the dpm in each fraction respective of the total dpm (sum of dpm in both fractions).

### Intestinal cholesterol absorption

Radiolabelled [^14^C]-cholesterol (2 x 10^−4^ μCi per mouse), in a bolus of olive oil, was administered orally by gavage (50 μl total volume) to control and *ob/ob* (n = 5 for each group) mice. Blood was collected at 6, 12 and 24 h and the plasma (25 μl volume) was liquid scintillation counted. Faeces were also collected from each animal, including the intestinal tract, after 24 h and was quantified for radiolabelled sterol.

### Quantitative PCR

The mRNA was extracted from liver and adipose tissue using the commercially available Qiagen RNeasy extraction kits (Qiagen, Hiden, Germany). The mRNA concentration was normalised to β-actin and cDNA were generated from the mRNA using Bio-rad iScript kits (Bio-rad Inc., Hercules, CA, United States). PCR was performed with each sample tested in triplicate and analysed using the 2^-∆∆CT^ method for relative gene expression (see S1 Table for primer sequences).

### Western blots of hepatic and adipose cholesterol transporters

Frozen liver and adipose tissue (~0.1 mg) were placed in RIPA buffer (with protease inhibitors and PMSF; Sigma) and homogenised using a Precellys 24 homogeniser (Bertin Technologies, Rockville, MD, USA). Protein concentrations were determined using the bicinchoninic acid assay (BCA) (Pierce, ThermoFisher Scientific, Waltham, MA, USA) kit and loaded onto 4–12% Bis-Tris Plus gels (Invitrogen, Carlsbad, CA, USA) at concentrations of 10–40 μg of protein. Standard running and blotting (iBlot Mini Stacks; Invitrogen) conditions were used. Primary antibodies SR-BI and ABCG1 are rabbit polyclonal (1:1000 and 1:500 dilution respectively) while ABCA1 and ABCG5 were mouse monoclonal (both used at 1:1000 dilution). All were sourced from Abcam (Cambridge, UK) except ABCG5 (Novus Biologicals; Littleton, CO, USA) while the secondary goat antibody (both anti-mouse and anti-rabbit) was from Novus (1:1000, Littleton, CO, USA).

### Precipitation of apoB lipoproteins

Whole serum was incubated with 20% polyethylene glycol (PEG-8000; Sigma) in 200mM glycine (ratio of PEG to serum volume = 2:5) for 20 minutes at room temperature before being centrifuged at 10,000 rpm for 30 minutes at 4 ^o^C to pellet the precipitate. The supernatants (apoB lipoprotein-depleted or HDL-fraction of the serum) were recovered and added to cells to measure efflux potential. Total (TC) and free cholesterol (FC) were determined using enzymatic colorimetric assays (Wako Chemicals, Richmond, VA, USA) to determine TC and FC concentration of whole serum and the HDL-fraction (apoB lipoprotein-depleted fraction) of the serum.

### Total cholesterol, triglyceride, free cholesterol, free glycerol and protein assays

TC, FC and triglyceride (TG) plasma/serum levels were determined using commercially available kits (Wako Chemicals, Richmond, VA, USA). Protein levels were quantified using the bicinchoninic acid assay (BCA) (Pierce, ThermoFisher Scientific, Waltham, MA, USA). Free glycerol in the plasma was determined using a commercially available kit (Cayman Chemicals, Ann Arbor, MI, USA).

### Mouse bone marrow-derived macrophages (BMDM)

L-cells were cultured and the media (RPMI 1640) harvested every 7 days as a source for macrophage colony-stimulating-factor for the bone marrow media. The L-cell conditioned media was centrifuged at 1000 rpm for 10 minutes, filtered (0.2 μm) and stored at -20^o^.

Femurs were dissected out and the bone marrow flushed out using a sterile wash media (DMEM, 5% FBS and P/S). The bone marrow was pelleted (1000 rpm, 5 minutes, room temperature) and resuspended with sterile bone marrow media (DMEM with 1 g/ml glucose, L-glutamine, 1.0 mM sodium pyruvate, 20% FBS, penicillin/streptomycin and 30% L-cell media v/v) and incubated for a further 5–10 days at 37^o^ C, 5% CO_2_, with a media change every 3 days, to allow differentiation of the cells to occur. BMDMs were then plated for cholesterol efflux experiments. For cholesterol-loading, BMDMs were incubated with an additional 100 μg/ml of acetylated LDL during the 24 h radiolabelling period.

### Statistical analysis

Results are expressed as the average ± SD. Significance between treatment groups and genotypes were determined using ANOVA and/or Student *t* test with a P value equal or less than 0.05.

## Results

### Plasma and liver lipids

Total cholesterol, LDL cholesterol and HDL-cholesterol concentrations were significantly higher in the *ob/ob* mice, compared to the wild type control animals (Table 1). Plasma total, HDL- and non-HDL triglycerides were also higher in the *ob/ob* mice but were not significantly different (Table 1). Liver TG levels were significantly higher in the *ob/ob* mice. No changes were detected in plasma glycerol levels.

**Table 1:** Plasma and liver lipids.

		Wildtype littermate	*Ob/ob*
**Plasma Lipids** **(mmol/L)**	**TC**	2.45 ± 0.10	4.20 ± 0.07*
	**non-HDLc**	0.91 ± 0.09	1.73 ± 0.08*
	**HDLc**	1.54 ± 0.12	2.47 ± 0.11*
	**TG**	1.086 ± 0.04	1.199 ± 0.03
**n = 6**	**non-HDL TG**	0.517 ± 0.03	0.646 ± 0.06
	**HDL TG**	0.455 ± 0.02	0.553 ± 0.04
	**Glycerol nmol/ml**	506 ± 57.16	448.3 ± 58.33
**Liver** **(mmol/l/μg protein)**	**TC**	0.80 ± 0.13	0.95 ± 0.25
**n = 4**	**TG**	1.50 ± 0.24	5.41 ± 0.34^a^

Plasma total cholesterol, HDL-c and non-HDL cholesterol concentrations, as well as plasma total triglyceride, free glycerol, HDL and non-HDL triglyceride were determined in 8-week old male *ob/ob* and wildtype littermate control mice using enzymatic methods. Liver TC and TG were determined from homogenised lysates using enzymatic kits and then normalised to protein concentrations. Values are mean ± S.E.M.

*p<0.0001

^a^p<0.0001 vs Wildtype littermate control mice.

### Body, liver and fat weights

The body mass of *ob/ob* male mice (n = 14) at 8 weeks of age was double that of their wildtype littermate controls (Control: 23.44 ± 0.54 g vs *ob/ob*: 45.25 ± 0.56 g, p<0.0001, S1A Fig). The *ob/ob* mice also had significantly higher liver weights than control mice (Control: 3.33 ± 0.39 g vs *ob/ob*: 1.11 ± 0.11 g, p<0.001, S1B Fig).

Body fat weight was determined by D_2_O and as expected, the *ob/ob* mice had significantly greater fat mass than the wildtype litter mate control mice (Control: 5.87±2.6 g vs *ob/ob*: 28.13±0.8 g, p<0.0001, S1C Fig).

### Reverse cholesterol transport

**Fig 1:**
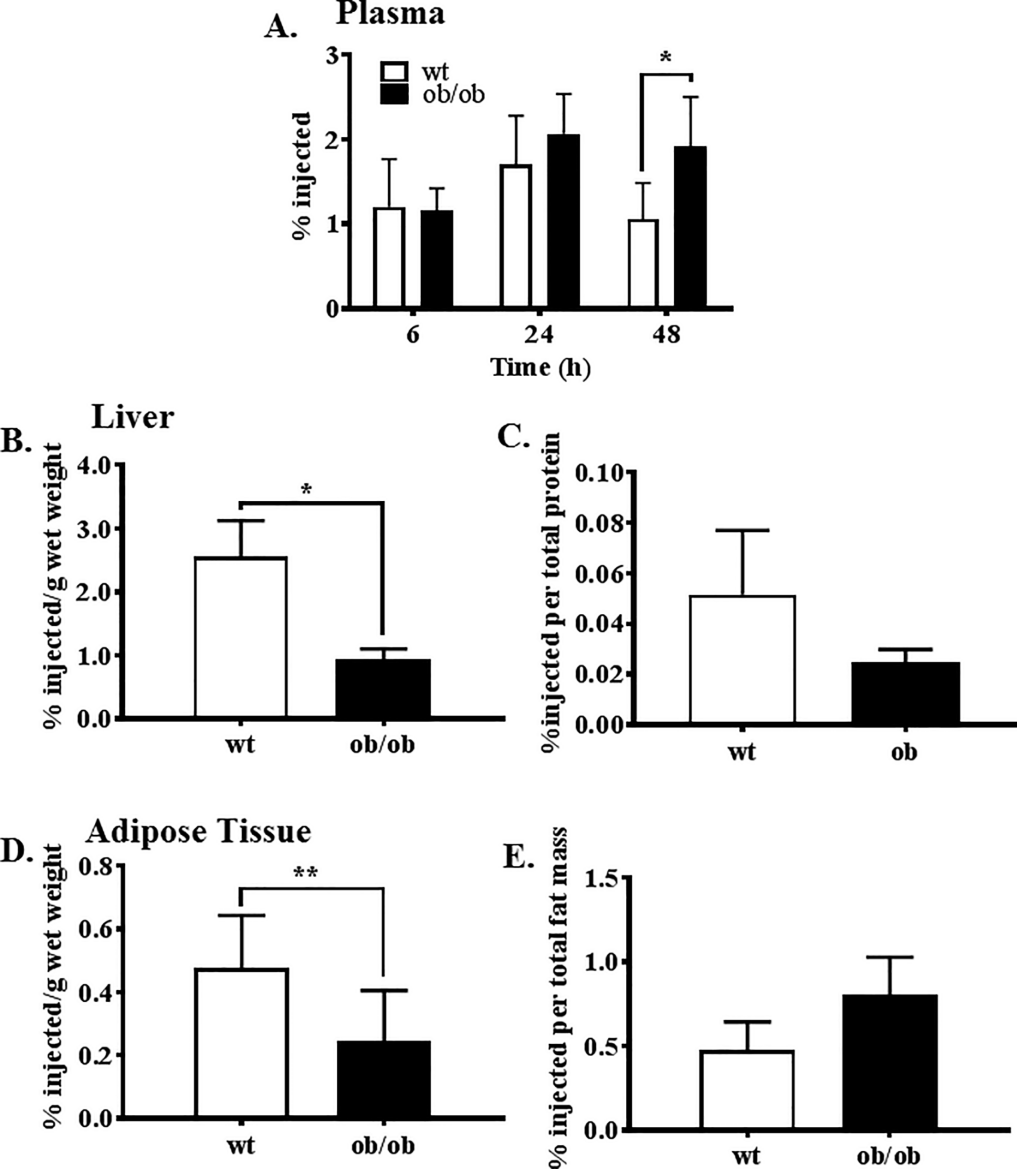
Radiolabelled cholesterol accumulation in the plasma, liver and adipose tissue. The concentration of radiolabelled cholesterol as percent of injected dose in (A) plasma over 48 h, (B) per gram of liver wet weight, (C) total liver protein, (D) per gram of adipose wet weight and (E) total adipose mass. A known volume of plasma (30 mL) was measured directly using liquid scintillation counting while lipids were extracted from tissue by Bligh-Dyer before quantifying. Values were then expressed as a % of the total amount of radiolabelled cholesterol injected. *p<0.0005, **p = 0.03.

*Ob/ob* mice (8 weeks old males; n = 14), when compared with littermate controls, had significantly more labelled cholesterol in the plasma at 48 h post-infusion (Fig 1A), indicating a slower clearance rate. [H^3^]-cholesterol accumulation in the liver was significantly lower in the *ob/ob* mice (Fig 1B). When total liver protein was taken into consideration no significant differences were observed between wildtype and *ob/ob* mice although there was a trend towards lower accumulation in the *ob/ob* liver (Fig 1C). Determination of cholesterol accumulation in the adipose tissue revealed there was significantly less cholesterol in the adipose tissue of *ob/ob* mice than in the wildtype littermate control mice (Fig 1D). No differences were observed between wildtype and *ob/ob* mice when total fat mass was accounted for (Fig 1E).

**Fig 2:**
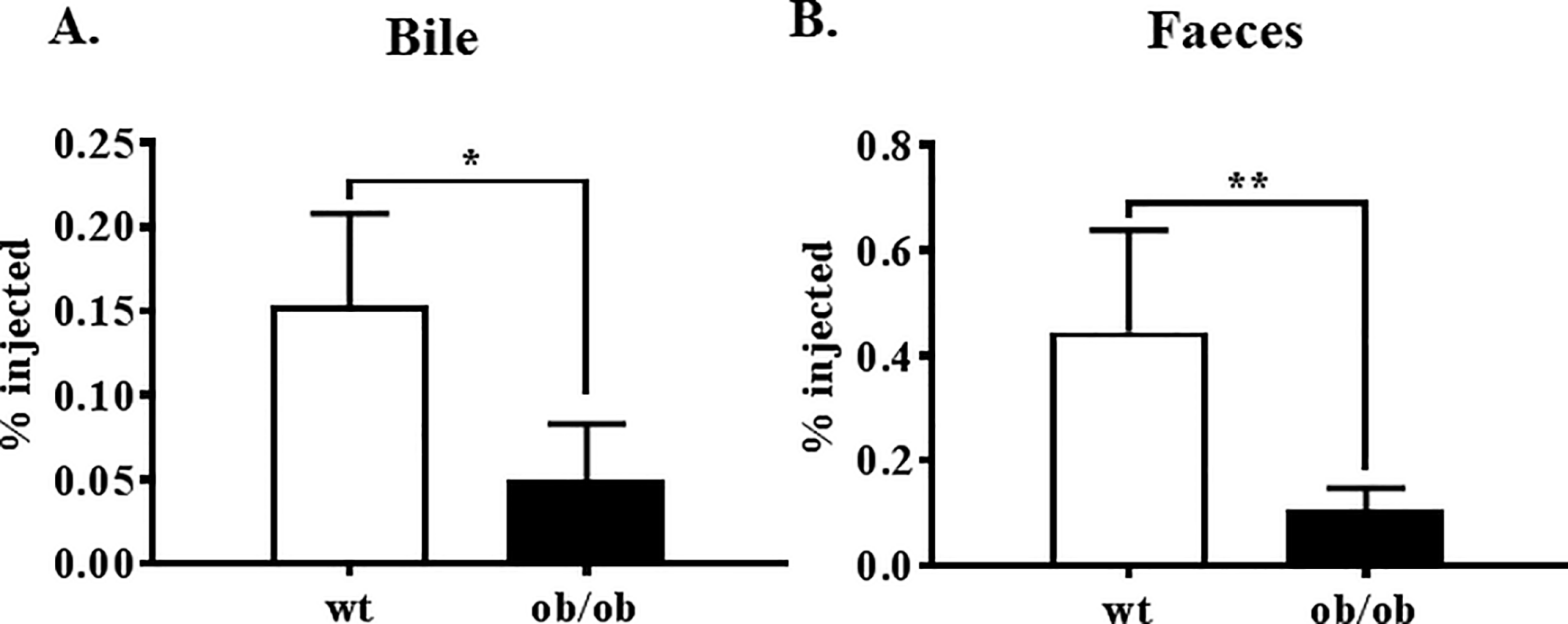
Radiolabelled cholesterol accumulation in the bile and faeces. The amount of cholesterol accumulation in the bile (A) was determined by measuring a known volume by liquid scintillation counting. Cholesterol accumulation in the faeces (B) as was determined by lipid extraction using the Bligh-Dyer method and then quantified by liquid scintillation counting. Both measures were then expressed as a % of the total amount of radiolabelled cholesterol injected. *p = 0.002; **p<0.0001.

We found significantly less labelled cholesterol in the bile (Fig 2A) and the faeces (Fig 2B) of *ob/ob* mice, when compared to the control mice.

### Intestinal cholesterol absorption and cholesterol-to-bile metabolism

There were no significant differences in the rate of cholesterol absorption from the intestine between the control and *ob/ob* mice. Labelled cholesterol concentrations were similar in the plasma (S2A Fig) and faeces (S2B Fig), up to 24 h following oral administration of [^14^C]-cholesterol. There was also no difference in the cholesterol-to-bile conversion between the mice (S3 Fig). It is interesting to note that over 90% of the radiolabelled cholesterol in the bile was in the BA fraction.

## Lipid transporter protein expression in liver and adipose

**Fig 3:**
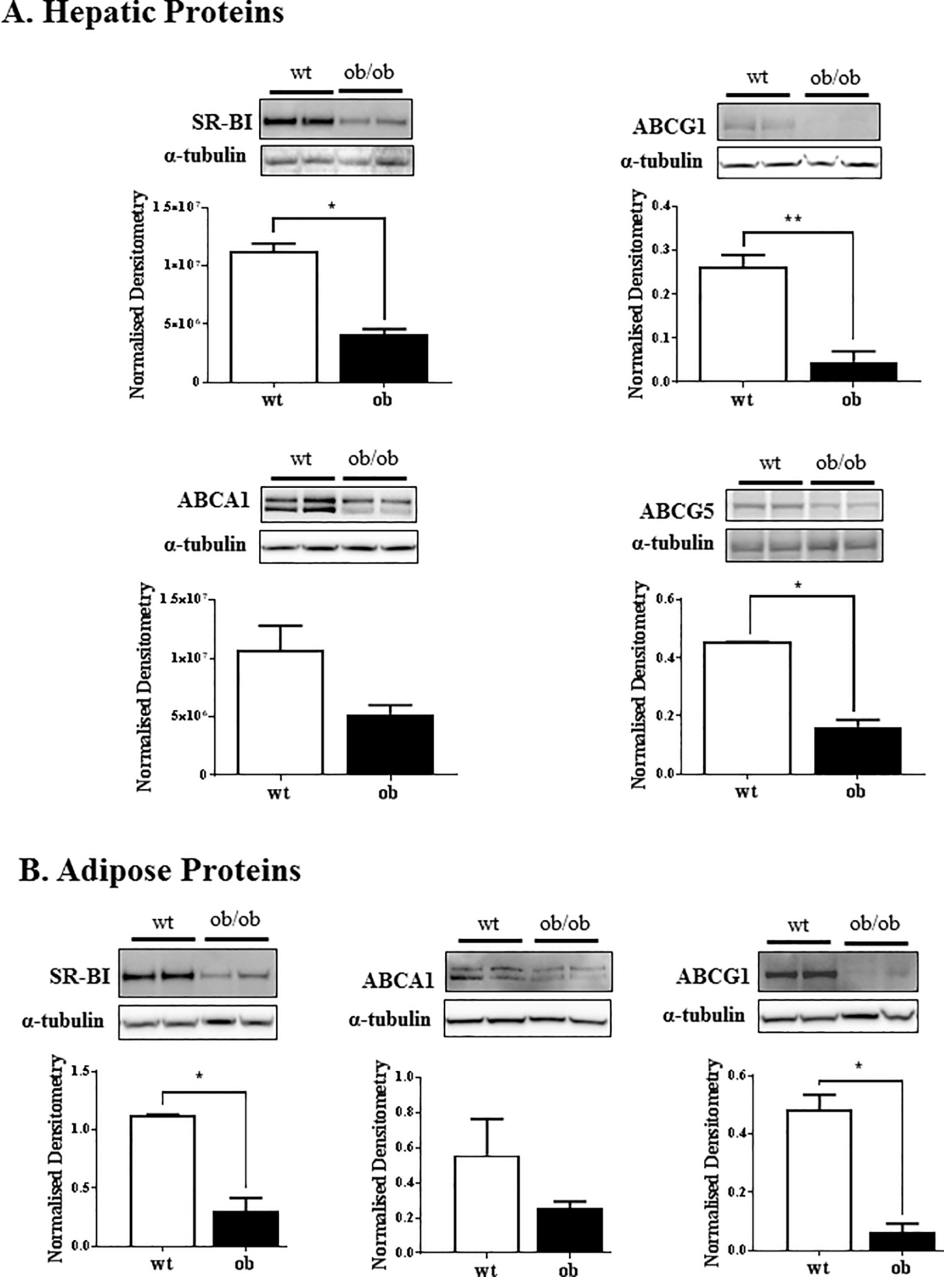
Western blot for SR-BI, ABCA1, ABCG1 and ABCG5 protein expression. Liver (A) and adipose (B) tissue were homogenised into lysates in RIPA buffer with protease inhibitors and PMSF. The lysates (10–20 μg protein for liver and 40 μg protein for adipose) were loaded onto a 3–12% SDS gel and transferred onto nitrocellulose membrane. The membranes were probed for SR-BI, ABCA1, ABCG1 and ABCG5 (liver only) and visualised with ECL and densitometry. Results are normalised to the α-tubulin loading control. n = 3–4 mice of each genotype; *p<0.01.

Liver and adipose lysates were quantified by Western blot for protein expression of SR-BI, ABCA1, ABCG1 and ABCG5 (liver only). The protein levels of hepatic SR-BI, ABCG1 and ABCG5 were significantly lower in the *ob/ob* mice than the control non-obese wildtype mice (Fig 3A). The protein expression of SR-BI and ABCG1 was also significantly lower in *ob/ob* adipose tissue compared to wildtype (Fig 3B).

### Cellular cholesterol efflux

**Fig 4:**
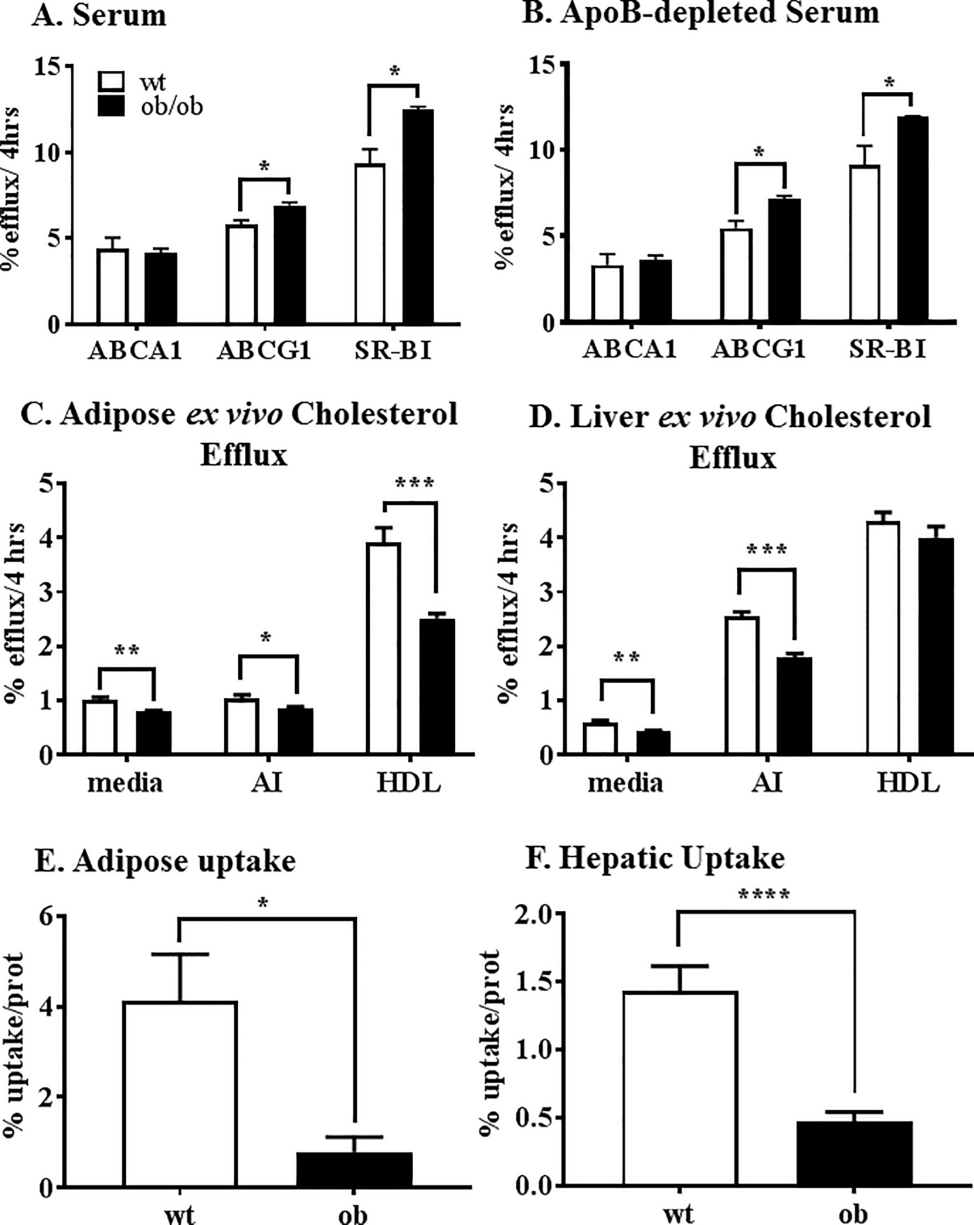
Cholesterol efflux potential of the serum and adipose and liver *ex vivo* cholesterol efflux capacity and cholesterol uptake of *ob/ob* and wt mice. Serum (A) was obtained from the controls (white bars) and *ob/ob* (black bars) mice, prior to the RCT procedure and used at 2.0% concentration on a set of cells expressed ABCA1, ABCG1 and SR-BI. The cells were prelabelled with [^3^H]-cholesterol and incubated with the serum for 4 h before the amount of radiolabel was determined in the media by liquid scintillation counting. Percent cholesterol efflux was determined as concentration of label in the media as a function of total label in the cells. Cellular cholesterol efflux was also quantified using the apoB-depleted fraction of the serum (B) where serum was incubated with PEG (2:5), pelleted and then the supernatant used at 2.4% v/v. Freshly isolated adipose (C) and liver tissue (D) were cut into small pieces, cholesterol-loaded with [^3^H]-cholesterol and incubated for 4 h with cholesterol acceptors apoA-I and HDL or media (passive) to measure adipose and liver *ex vivo* efflux capacity. Freshly isolated adipose (E) and liver tissue (F) were also used to measure cholesterol uptake by measuring the amount of [^3^H]-cholesterol taken up over a 24 h period. Lipids were extracted from the tissue with isopropanol and [^3^H]-cholesterol quantified by liquid scintillation counting, which was then normalised to protein concentration in each well. RCT, reverse cholesterol transport; SR-BI, scavenger receptor class B type 1; ABCG1, ATP-binding cassette sub-family G member 1; PEG, polyethylene glycol. *p<0.05; **p<0.002; ***p<0.00005;****p<0.003.

Determination of the cellular cholesterol efflux potential of *ob/ob* and control mouse whole serum was measured. No changes were found in the ABCA1-mediated cellular efflux potential of the serum of wildtype and *ob/ob* mice (Fig 4A). However, *ob/ob* serum induced significantly greater ATP-binding cassette sub-family G member 1 (ABCG1)-mediated cellular efflux and scavenger receptor class B type 1 (SR-BI)-mediated cellular efflux compared to the control serum.

The HDL-fraction of the serum, obtained after PEG precipitation, in which the apoB lipoproteins are precipitated and removed, showed a similar pattern as the whole serum (Fig 4B). *Ob/ob* apoB-depleted serum, ABCG1-mediated cellular cholesterol efflux was higher as was SR-BI- mediated cellular cholesterol efflux. There were no changes in ABCA1-mediated cholesterol efflux between groups.

### Liver and adipose *ex vivo* cholesterol efflux

Adipose (Fig 4C) and liver (Fig 4D) *ex vivo* cholesterol efflux were performed, where the fresh tissue was cut into small pieces and radiolabelled-cholesterol efflux movement to apoA-I and HDL or media (passive movement) was determined. Passive cholesterol diffusion to the media and apoA-I-mediated cholesterol efflux was significantly lower from adipose and liver tissue of *ob/ob* mice, compared to wildtype mice. *Ob/ob* adipose tissue also had significantly lower HDL-mediated cholesterol efflux reflecting impairment of either SR-BI, ABCG1 or both.

### Hepatic and adipose cholesterol uptake

Adipose (Fig 4E) and liver (Fig 4F) uptake of radiolabelled-cholesterol was also quantified. The uptake of radiolabelled-cholesterol into adipose tissue and liver of *ob/ob* mice was significantly lower than wildtype control animals.

### Real-time PCR

*m*RNA was isolated from liver tissue and the expression of *abca1*, *abcg1*, *abcg5* and *sr-b1* were quantified by real-time PCR (S1 Table). Whilst there were no significant changes in the hepatic expression of *abca*1 or *abcg*5, there was significant 4-fold increase in the hepatic *m*RNA expression of both *abcg*1 and *sr-b1* in the *ob/ob* mice, compared to the control animals (p<0.01; S4A Fig). *Sr-b1*, *abca1* and *abcg1 m*RNA levels in adipose tissue were also determined (S4B Fig). No significant changes were detected.

### BMDM cellular cholesterol efflux

**Fig 5:**
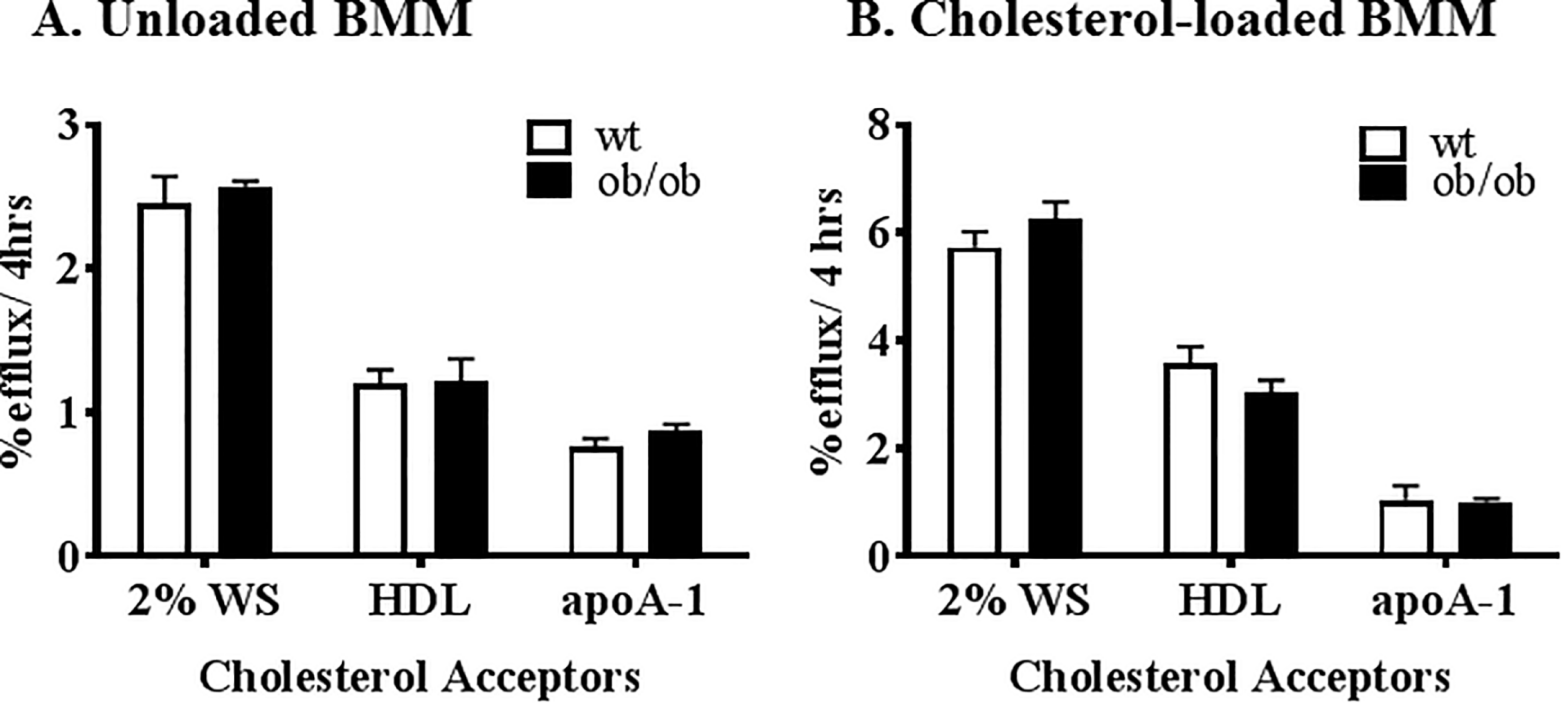
Cellular cholesterol efflux from bone marrow-derived macrophages. The bone marrow of the littermate (white bars) and *ob/ob* (black bars) animals was harvested from the femur of 6 week old male mice and differentiated into macrophages using L-cell media. The cholesterol effluxing ability of the BMM were quantified using 2.0% whole serum, HDL (50 mg/ml) and apoA-I (10 mg/ml). The BMDM were tested when unloaded (A) and also after loaded (B) with addition of 100 mg/ml of acLDL during the radiolabelling period of the cells. BMDM, bone marrow-derived macrophages; acLDL, acetylated LDL.

The cholesterol efflux capabilities of BMDMs from *ob/ob* and wild type littermate mice were assessed from either: a standard sample of 2% pooled whole human serum, apoA-I (10 μg/ml) and HDL (50 μg/ml). No changes were found in cholesterol efflux capacity between wildtype control and *ob/ob* BMDMs for either unloaded cholesterol (Fig 5A) or cholesterol loaded (+acLDL, Fig 5B) BMDMs. The absolute FC and CE values were different between the *ob/ob* and control BMDM after cholesterol loading (23.2 ± 2.5 vs 10.34 ± 1.23 μg/mg protein for FC and 7.47 ±1.05 vs 1.39 ±0.36 μg/mg protein for CE respectively), however, the ratio (FC:CE) of fold change were similar indicating the handling and distribution were similar for the different genotypes.

## Discussion

In this study, a genetic mouse model of obesity was used to investigate the effect of adiposity on RCT. Significant weight gain in the *ob/ob* mouse was associated with an impairment in RCT. Labelled cholesterol from injected macrophages was not cleared from the plasma as efficiently as control animals. Furthermore, livers and adipose tissue from *ob/ob* mice had an impaired ability to take up the labelled cholesterol and to efflux it. The protein expression of the lipid transporter ABCA1, ABCG1 and SRB1 was lower in the liver and adipose tissue of *ob/ob* mice, compared to the non-obese wildtype controls. There was, however, a substantial 60% increase in HDL-cholesterol in the *ob/ob* mice. Consistent with this, serum and apoB-depleted plasma from *ob/ob* mice induced significantly more ABCG1 and SR-B1-mediated cholesterol efflux than controls, demonstrating that this part of the RCT pathway was not impaired. Taken together, our findings suggest that in obesity, there is an increased retention of cholesterol in the circulation due to less hepatic and adipose tissue uptake and may explain, at least in part, the increase in cardiovascular disease in patients with obesity.

**Fig 6:**
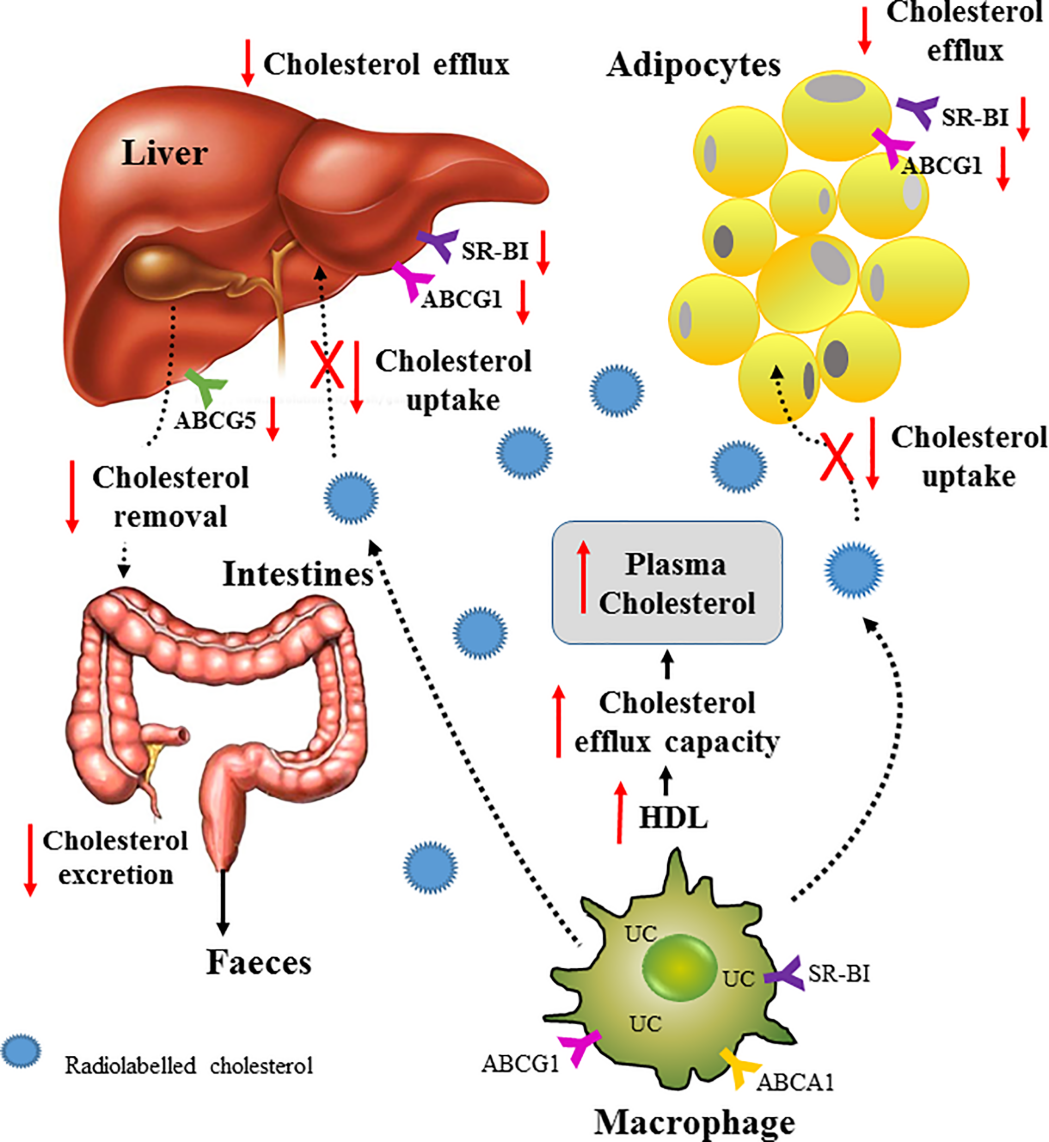
Schematic of RCT in the *ob/ob* mouse. The movement of cholesterol from the macrophage into blood is via ABCA1-, ABCG1- and SR-BI-mediated cholesterol efflux and this is unaffected in the *ob/ob* mice. Transport of the cholesterol throughout the body is mainly done by lipoproteins and in particular, HDL. However, uptake of cholesterol by the liver and adipocytes is compromised resulting in longer retention time in the blood and slower clearance of the cholesterol from the body via the bile and faeces. The HDL and serum of *ob/ob* mice have enhanced cholesterol efflux capacity which may have contribute further to the increase in plasm cholesterol.

RCT is comprised of a number of mechanisms including cellular cholesterol efflux to HDL, uptake of HDL by the liver, the conversion of cholesterol into bile and its excretion into the intestine to be removed in the faeces. The protocol used in this study addresses macrophage-specific RCT as the movement of labelled cholesterol is followed from injected macrophages [21]. In the *ob/ob* mice, it was observed that the atheroprotective mechanism of RCT is compromised as labelled cholesterol is increased in the plasma of *ob/ob* mice, suggesting reduced clearance. The uptake of HDL cholesterol by the liver is a critical step in the RCT pathway. This study showed directly that cholesterol uptake by both the liver and adipose tissue were significantly impaired (Fig 6). The liver expresses the highest amount of total tissue ABCG1 and SR-B1 protein, which play a major role in HDL cholesterol uptake [22]. The current study found that *ob/ob* mice had significantly reduced protein expression levels of hepatic ABCG1 and SR-B1 (Fig 3A) which is consistent with our finding of reduced efflux of cholesterol from the liver. The livers of the *ob/ob* mice were more than double the weight of the wildtype control livers and it was noted that they were also pale in colour, indicating a high lipid content. It is possible that the hepatocytes of the *ob/ob* mice are already excessively loaded with lipid and therefore unable to take up the labelled cholesterol. Evidence for this has been found in macrophages in which it was shown that free cholesterol-loading causes a defect in cholesterol uptake via increase degradation of ABCA1 [23]. Consistent with this, we found that the *ex vivo* uptake of cholesterol into the liver and adipose tissue was significantly impaired in the *ob/ob* mice.

As the labelled cholesterol was restricted in its ability to be taken up by the liver, it follows that less will be metabolised to bile acids and then the faeces. The current study found that there was significantly less labelled cholesterol in both the bile and the faeces. The expression of ABCG5 in the *ob/ob* liver was significantly less than the controls. ABCG5 complexes with ABCG8 to transport biliary excretion of cholesterol from the liver and are expressed and regulated in a co-ordinated fashion [24]. As only small amounts of labelled cholesterol is taken up by the liver of *ob/ob* mice, the transport requirements for biliary cholesterol from the liver is reduced and thereby it follows that there was is a decrease in hepatic ABCG5.

Another key part of the RCT pathway is the efflux of cholesterol from peripheral tissues to HDL. This part of the RCT pathway was not impaired in the *ob/ob* mice as the SR-B1 and ABCG1-mediated cholesterol efflux capacity of both serum and apoB-depleted plasma was actually higher. This is likely due to the elevation in HDL-cholesterol levels, a previously reported genetic characteristic of this genotype [25]. These findings also indicate that HDL from *ob/ob* mice is not dysfunctional in terms of mediating cholesterol efflux and is consistent with previous studies [26]. Although, in the context of obesity it is perhaps not surprising that excessively lipid loaded cells in the periphery would be primed to try and off-load more cholesterol, than normo-lipidemic cells. An enhancement of cholesterol efflux, in the case of the *ob/ob* mice, is likely to have in fact added to the plasma cholesterol pool as the next step in the RCT pathway of hepatic cholesterol uptake is significantly impaired, effectively trapping the cholesterol in the circulation.

Another factor that may affect RCT is cholesterol reabsorption in the intestine [27]. Cholesterol and bile are secreted from the liver and into the gut where it is taken up in the ilium and the cholesterol is recycled. This part of the RCT pathway did not appear to be altered in the *ob/ob* mice. Following a bolus dose of [^14^C]-cholesterol, a similar amount of labelled cholesterol was absorbed into the blood and the faeces of both the *ob/ob* and the wildtype littermate mice.

Previous studies have shown that adipocytes are able to efflux cholesterol to apoA-I and HDL via ABCA1 and SR-BI [28]. This would suggest that adipocytes also play a role in RCT. In support of this, it has been reported that HDL cholesterol esters can be taken up by adipocytes for energy storage [29,30]. It may have been expected therefore that the significantly higher amounts of adipose tissue in the *ob/ob* mice could have helped to clear the labelled cholesterol from the circulation. This was not, however, the case. Similar to the liver, the adipocytes of *ob/ob* mice were found to have an impaired ability to take up cholesterol from the circulation, likely due to an already present high lipid content with very little remaining capacity to take up more cholesterol. In this study, the adipose tissue from the obese mice were also shown to have significantly reduced ability to efflux cholesterol. Consistent with this the protein levels of ABCA1, ABCG1 and SRB1 were lower in the *ob/ob* adipose tissue. The combination of reduced cholesterol uptake and cholesterol efflux into and from the liver and adipose explains the decreased clearance of cholesterol in the plasma and decrease in RCT efficiency.

Bone marrow was harvested and differentiated into macrophages to determine whether there were differences in macrophage cholesterol efflux capacity due to the genetic deficiency of leptin or the induction of obesity. No changes were observed in the ability of BMDMs to efflux cholesterol to whole serum, HDL or apoA-I between *ob/ob* and wildtype control mice. We also showed that the BMDMs had similar ability to handle and distribute cholesterol into the different intracellular cholesterol pools. This shows that the impairment in RCT in the *ob/ob* mice cannot be attributed to changes in macrophage efflux capacity.

Due to its phenotype [31] and lipid profile, *ob/ob* mice are unable to develop atherosclerosis however, in an atherogenic background such as LDLr knockout, the double knockout has a plaque area that greatly exceeds (more than 4-fold) that of the control LDLr knockout [26]. The finding in this study where increased fat mass plays a role in reducing RCT may account for this greatly increased atherogenicity in the double knockout.

In conclusion, this study has demonstrated for the first time that obesity impairs RCT. The limiting step in the RCT pathway appears to be a decline in the ability to take up cholesterol into the liver and adipose tissue and well as an impaired ability to efflux the cholesterol. Impaired RCT and retention of cholesterol in the plasma may provide, in part, an explanation for the increase in cardiovascular disease in patients with obesity.

## Supporting information

S1 Fig Differences in body and tissue mass between the ob/ob and wild-type littermates. Total body weight (A) and wet liver weight (B) was measured at the 48 h timepoint of RCT and showed that ob/ob (black squares) had significantly greater body and liver weight than the control littermates (black circles) (*p<0.0001). Total fat mass (C) was determined by the distribution of injected heavy water after 3 h, by mass spectrometry, and determined as the difference between total body weight and total body water. **p<0.005.
(PPTX)

S2 Fig Intestinal cholesterol reabsorption efficiency between the ob/ob and wild type littermate control. Cholesterol reabsorption was determined from (A) plasma at 6, 12 and 24 h and (B) the faeces (24 h) after [14C]-cholesterol was orally administered with a bolus of olive oil (50 ml). The plasma was counted directly by liquid scintillation counting while lipids was extracted from the faces by Bligh-Dyer. Littermate controls (open circles); ob/ob (black squares).
(PPTX)

S3 Fig Labelled cholesterol content in bile acid and neutral sterol fractions of the bile. The compositional end point of the radiolabelled cholesterol injected into the mice after 48 h. The bile was collected and bile acids (BA) where separated from the neutral sterol using petroleum ether. The two fractions were scintillation counted and determined as percent of total counts. Vales are mean ± SEM.
(PPTX)

S4 Fig Tissue mRNA expression of *abca1*, *abcg1*, *srb1* and *abcg5*. Liver (A) and adipose (B) *m*RNA was isolated using standard protocols and the PCR was performed using standard cyclic conditions. Littermate control *m*RNA levels was set as 1.0. *p<0.01.
(PPTX)

S1 Table Primer sequences used for RT-qPCR. (PPTX)

## Abbreviations

RCT: reverse cholesterol transport
BMDM: bone marrow-derived macrophages
HDL-c: high density lipoprotein cholesterol
P/S: penicillin and streptomycin
TC: total cholesterol
FC: free cholesterol
BCA: bicinchoninic acid assay
D_2_O: heavy water or deuterium oxide
*m*RNA: messenger RNA
SR-BI: scavenger receptor class B type 1
ABCG1: ATP-binding cassette sub-family G member 1
PEG: polyethylene glycol
ABCG8: ATP Binding Cassette Subfamily G Member 8
ABCG5: ATP Binding Cassette Subfamily G Member 5
acLDL: acetylated LDL
