# Supplementary figures and images for: Induction of obesity impairs reverse cholesterol transport in ob/ob mice
Source: PLoS One. 2018 Sep 14;13(9):e0202102. doi: 10.1371/journal.pone.0202102 (PMC6138368; doi:10.1371/journal.pone.0202102)

## Slide 1
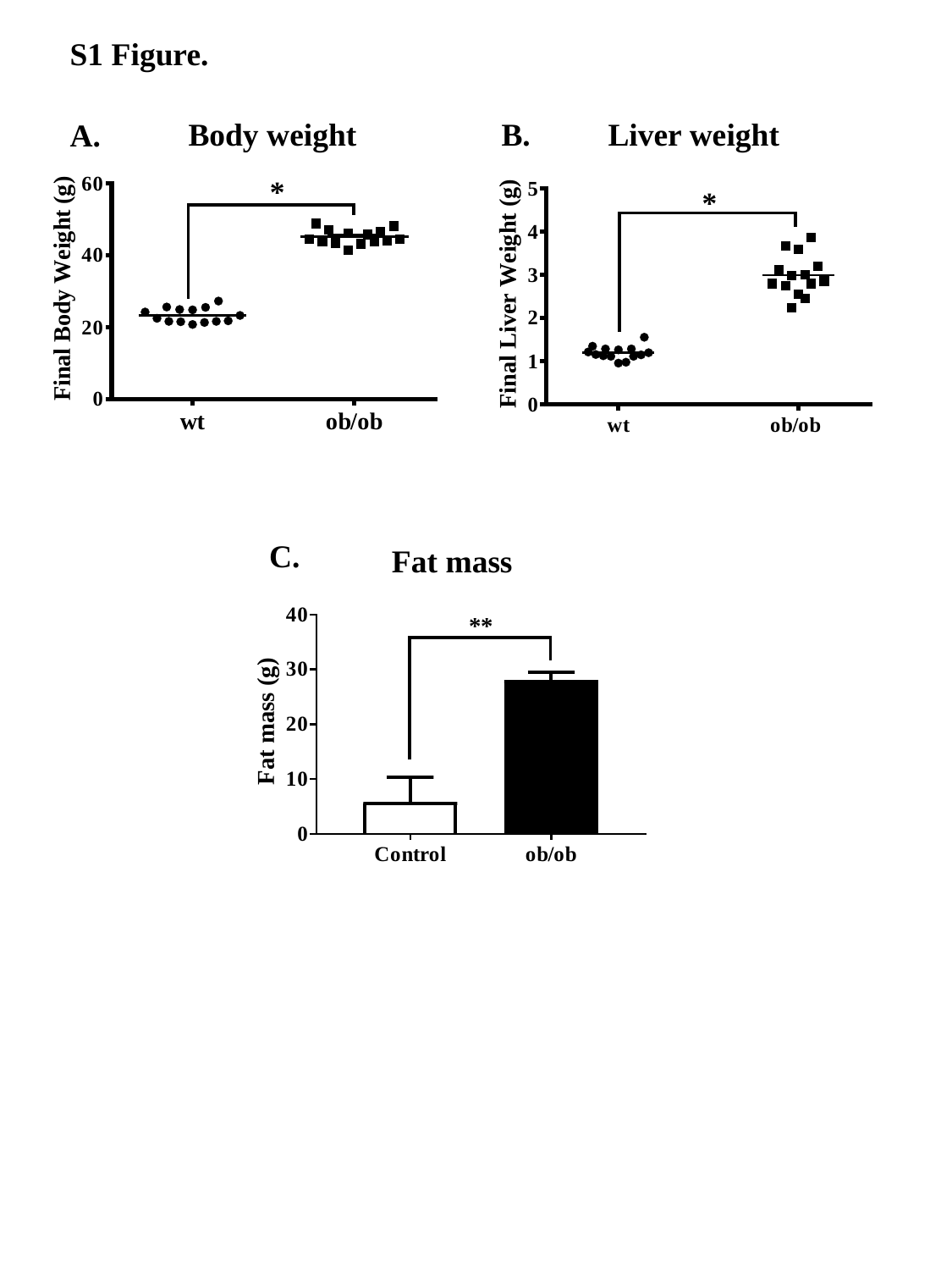

S1 Figure.
B.
Liver weight
Body weight
A.
 C.
Fat mass

Supplement: S1 Fig — Total body weight (A) and wet liver weight (B) was measured at the 48 h timepoint of RCT and showed that ob/ob (black squares) had significantly greater body and liver weight than the control littermates (black circles) (*p<0.0001). Total fat mass (C) was determined by the distribution of injected heavy water after 3 h, by mass spectrometry, and determined as the difference between total body weight and total body water. **p<0.005. (PPTX) [file pone.0202102.s001.pptx]

## Slide 1
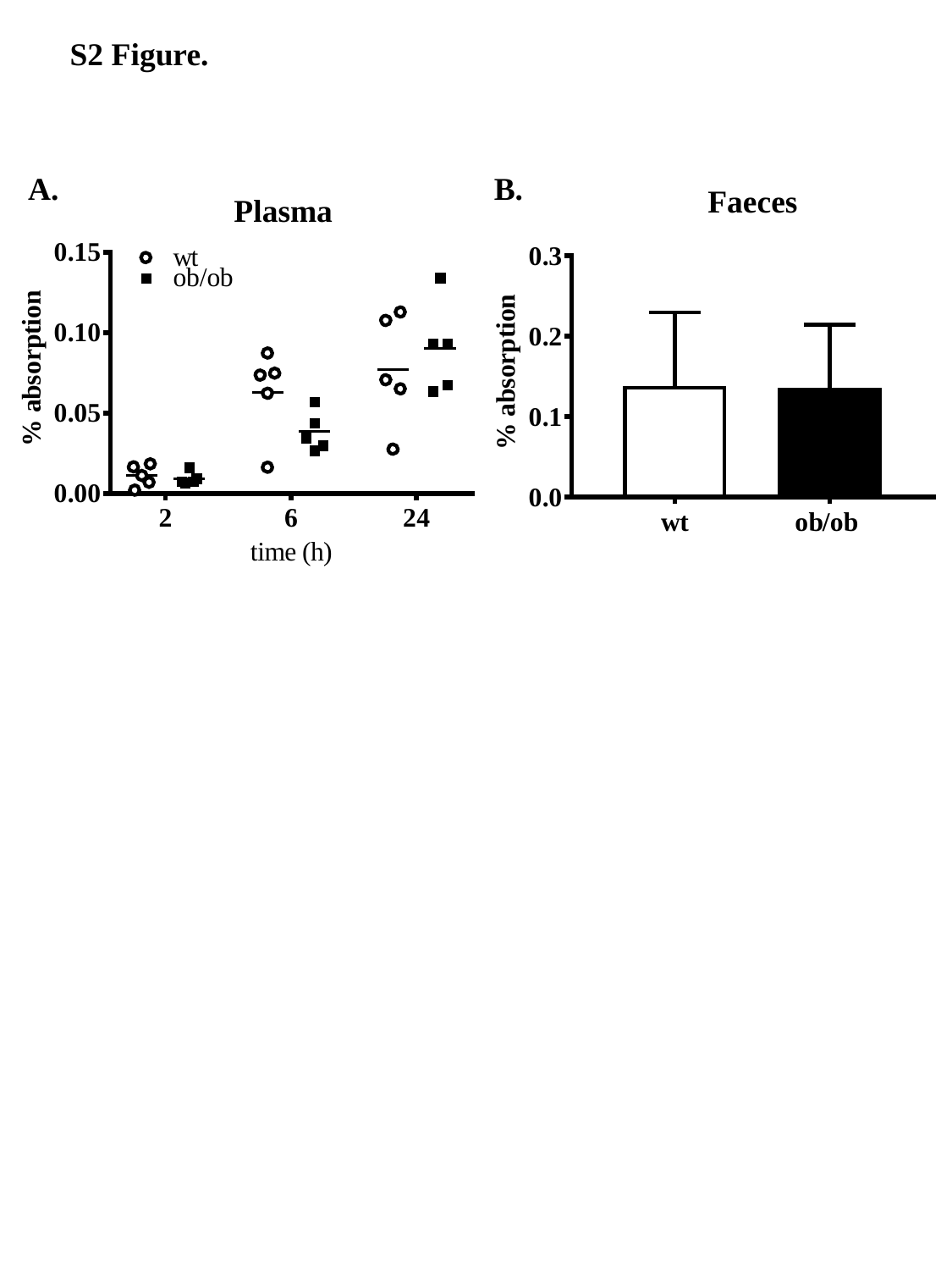

S2 Figure.
A.
B.
Faeces
Plasma

Supplement: S2 Fig — Cholesterol reabsorption was determined from (A) plasma at 6, 12 and 24 h and (B) the faeces (24 h) after [14C]-cholesterol was orally administered with a bolus of olive oil (50 ml). The plasma was counted directly by liquid scintillation counting while lipids was extracted from the faces by Bligh-Dyer. Littermate controls (open circles); ob/ob (black squares). (PPTX) [file pone.0202102.s002.pptx]

## Slide 1
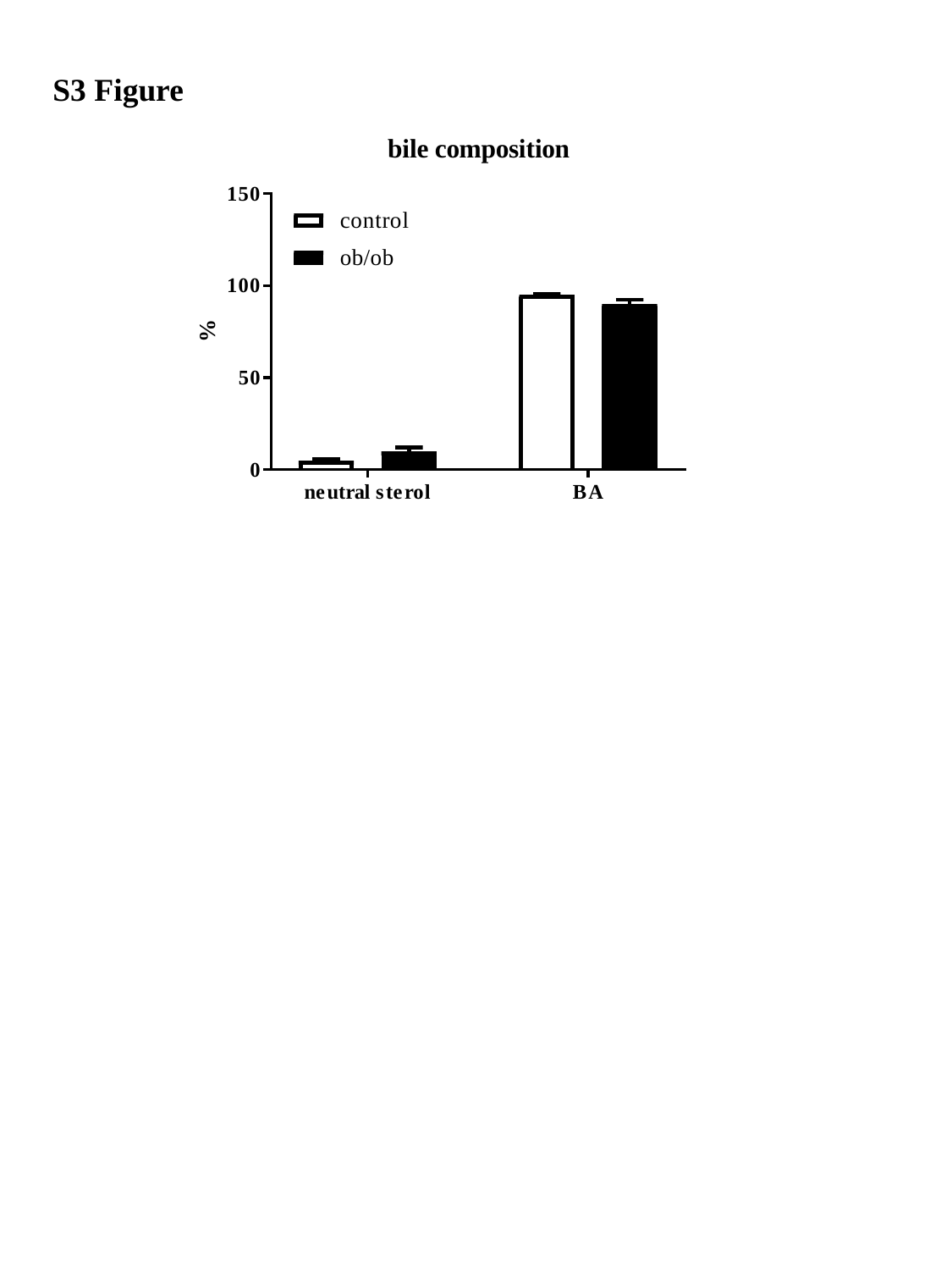

S3 Figure

Supplement: S3 Fig — The compositional end point of the radiolabelled cholesterol injected into the mice after 48 h. The bile was collected and bile acids (BA) where separated from the neutral sterol using petroleum ether. The two fractions were scintillation counted and determined as percent of total counts. Vales are mean ± SEM. (PPTX) [file pone.0202102.s003.pptx]

## Slide 1
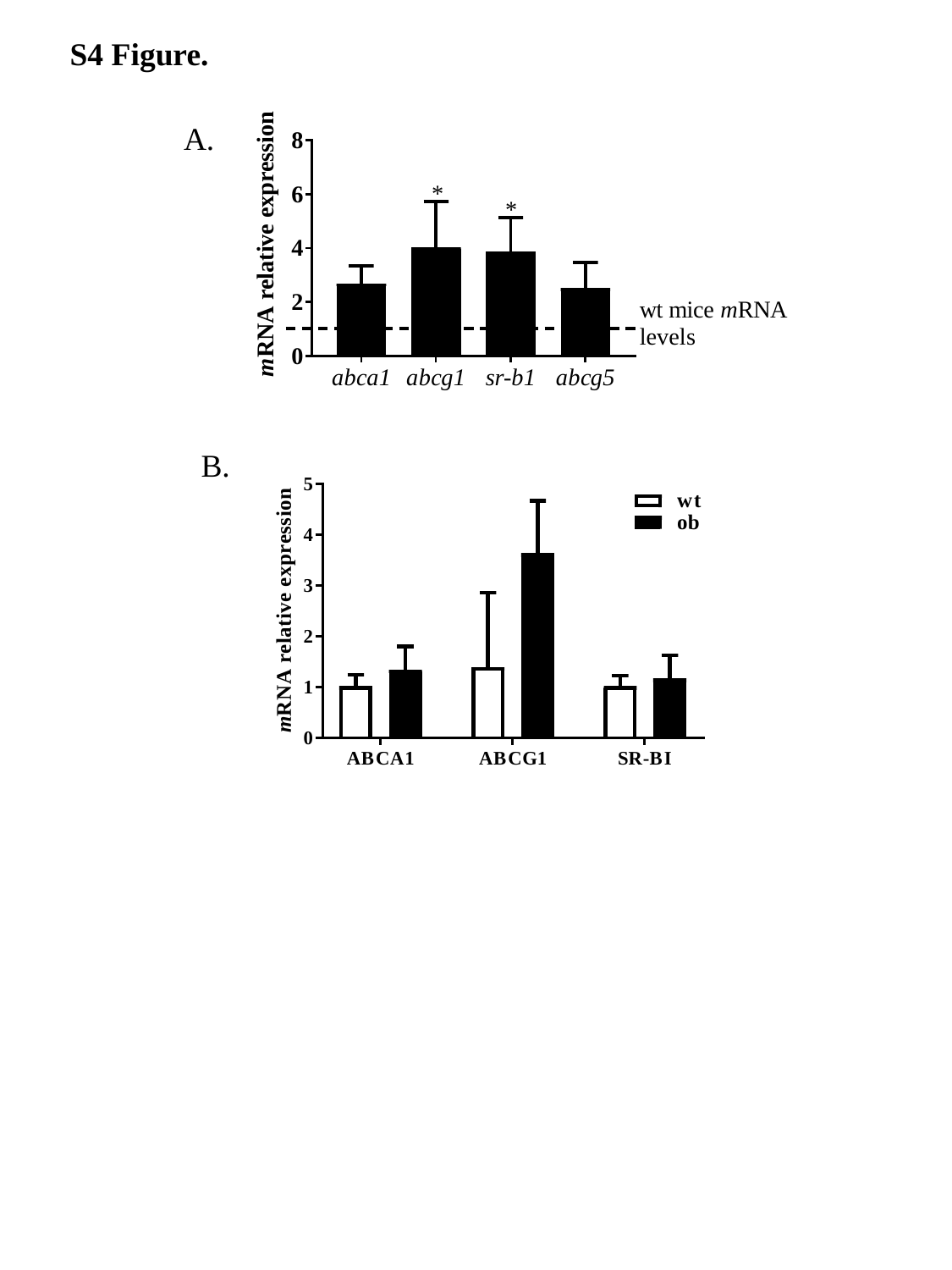

S4 Figure.
A.
B.

Supplement: S4 Fig — Liver (A) and adipose (B) mRNA was isolated using standard protocols and the PCR was performed using standard cyclic conditions. Littermate control mRNA levels was set as 1.0. *p<0.01. (PPTX) [file pone.0202102.s004.pptx]

## Slide 1
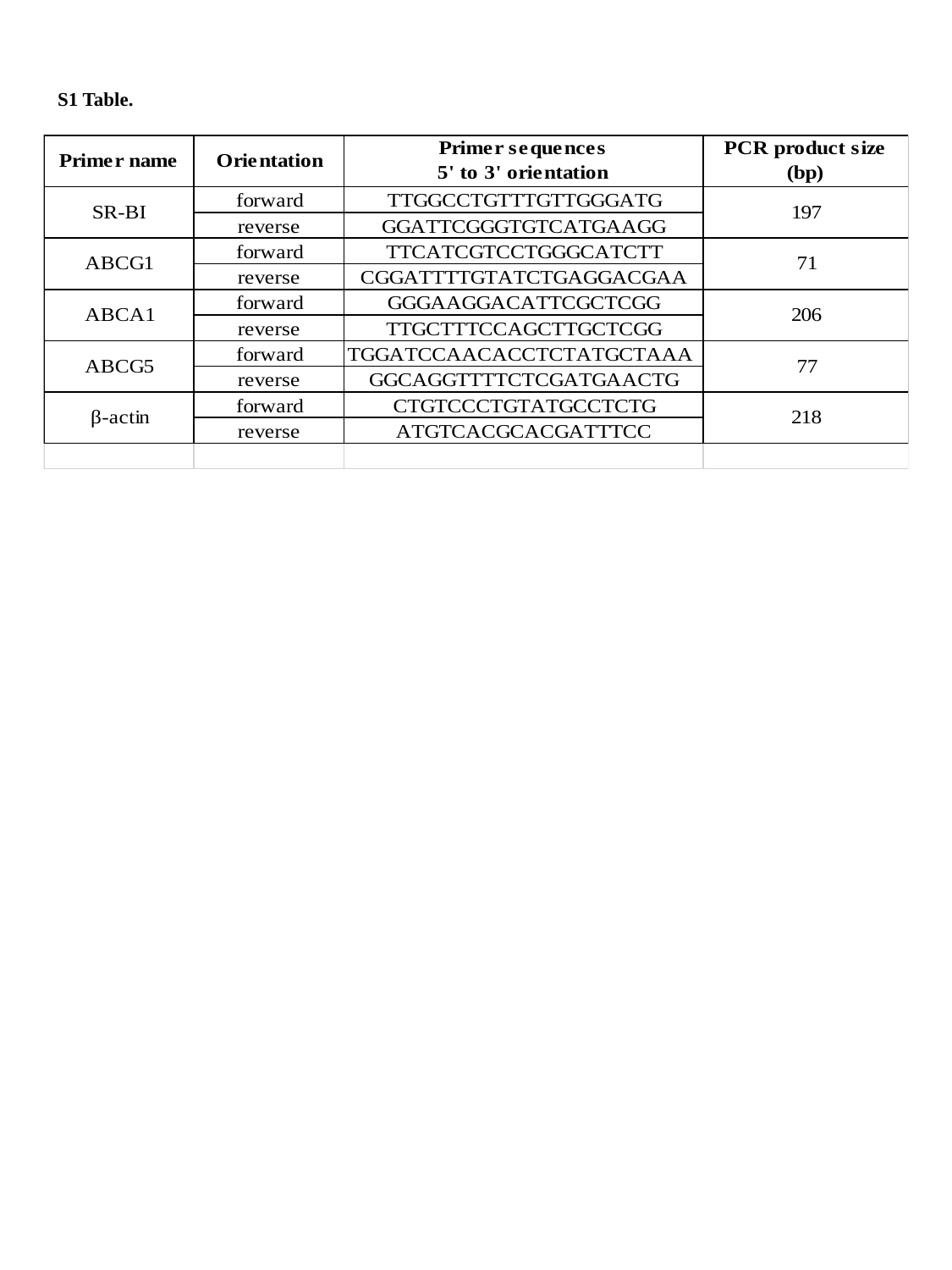

S1 Table.

Supplement: S1 Table — (PPTX) [file pone.0202102.s005.pptx]
